# The Local Coordination Effects on the Reactivity and Speciation of Active Sites in Graphene-Embedded Single-Atom Catalysts over Wide pH and Potential Range

**DOI:** 10.3390/nano12234309

**Published:** 2022-12-05

**Authors:** Milica S. Ritopečki, Ana S. Dobrota, Natalia V. Skorodumova, Igor A. Pašti

**Affiliations:** 1 University of Belgrade—Faculty of Physical Chemistry, Studentski trg 12-16, 11158 Belgrade, Serbia; 2 Department of Materials Science and Engineering, School of Industrial Engineering and Management, KTH—Royal Institute of Technology, Brinellvägen 23, 100 44 Stockholm, Sweden

**Keywords:** single-atom catalysts, graphene, activity, reactivity, stability, Pourbaix plots

## Abstract

Understanding the catalytic performance of different materials is of crucial importance for achieving further technological advancements. This especially relates to the behaviors of different classes of catalysts under operating conditions. Here, we analyzed the effects of local coordination of metal centers (Mn, Fe, Co) in graphene-embedded single-atom catalysts (SACs). We started with well-known M@N_4_-graphene catalysts and systematically replaced nitrogen atoms with oxygen or sulfur atoms to obtain M@O_x_N_y_-graphene and M@S_x_N_y_-graphene SACs (x + y = 4). We show that local coordination strongly affects the electronic structure and reactivity towards hydrogen and oxygen species. However, stability is even more affected. Using the concept of Pourbaix plots, we show that the replacement of nitrogen atoms in metal coordinating centers with O or S destabilized the SACs towards dissolution, while the metal centers were easily covered by O and OH, acting as additional ligands at high anodic potentials and high pH values. Thus, not only should local coordination be considered in terms of the activity of SACs, but it is also necessary to consider its effects on the speciation of SAC active centers under different potentials and pH conditions.

## 1. Introduction

The current energy crisis has put a focus on the development of novel efficient materials for energy conversion. Electrocatalysis is expected to play a key role in this direction, as it can offer many solutions in the energy race. However, electrocatalysts need to be active, stable, and affordable, facing us with the fact that no perfect solution currently exists. 

One of the key strategies for lowering catalyst price is to reduce the size of the particle, ideally to nano dimensions, thus increasing the surface-to-volume ratio and number of active sites exposed to the reaction medium. Even in this case, the cores of nanoparticles are not active, which is a waste of active components. Moreover, the use of precious metal-based catalysts also significantly impacts the price. However, a relatively novel class of catalysts, single-atom catalysts (SACs), alleviates this issue since the active site consists of only one atom. Furthermore, the active atom is embedded in a matrix that significantly impacts the activity of the active center, allowing alteration of SAC reactivity compared to nanosized or bulk counterparts [1,2,3].

SACs are usually experimentally characterized using techniques that provide resolution at the atomic level, including high-resolution transmission electron microscopy (to confirm the presence of single atoms), synchrotron-based techniques such as XANES (to address local coordination), and other surface-sensitive techniques, such as XPS (to observe the oxidation state of single atoms) [4]. While some of these techniques can be applied in situ or in operando, most studies consider that the structure of the SAC active site is the same as that observed under high vacuum conditions. Nevertheless, electrocatalytic processes typically take place under severe conditions. For extended surface and nanosized catalysts, it is well-accepted that surface electrochemical processes take place, such as the adsorption of different spectating species, which affects catalyst performance [5,6,7]. Thus, there is no reason to expect such processes not to occur at SACs used in electrocatalysis. In fact, some recent experimental and theoretical studies have suggested that the adsorption of different species at active sites of SACs leads to altered catalytic activity, selectivity, and stability [8,9,10,11,12], or even changes in the nature of the active site in different media (pH) [13]. The mentioned works are particularly related to cases of M-N_4_ SACs where the metal center is embedded in a carbon lattice and coordinated by four nitrogen atoms. The class of M-N_4_ SACs has received increased attention because it has turned out to be a rather promising replacement for platinum-based catalysts, while also showing high catalytic activity in other reactions [14,15,16,17]. The most interesting metals in this sense were found to be Fe, Co, and Mn [18,19,20].

While it is natural to expect that some adsorption processes can take place on the SAC active center, there are two important facts to consider. First, such processes are typically disregarded and SACs are considered to be electrode potential- and pH-independent systems identical to those observed under vacuum conditions. Second, an adsorbate attached to the SAC active site creates a whole new active site. Namely, in the case of extended surface catalysts and nanoparticles, there are ensembles of active sites. Adsorbed species usually act as spectators occupying a certain number of active sites, thus reducing the activity but a large electronic effect is usually not present [21]. However, in the case of SACs, once an adsorbate/ligand attaches to the active center, it is completely changed. Hence, as the matrix cannot be separated from the active metal atoms, the same holds true for any adsorbate/ligand attached to the original active site [22]. 

In the present work, we complement our previous work exploring the stability of SACs [23], analyzing the case of M-N_4_ (M = Mn, Fe, Co) centers where the metal coordination sphere is altered by systematically replacing nitrogen atoms with sulfur or oxygen atoms. To investigate stability, we calculated a series of standard electrode potentials for different reactions considered at the active centers and used them to construct Pourbaix diagrams [24,25,26] for selected cases. Furthermore, for the case of hydrogen evolution reaction (HER), we show that it is necessary to consider the stability under operating conditions before making any particular conclusions about HER activity. Finally, we discuss possible strategies to experimentally observe SAC active site alterations under electrochemical conditions, which are more affordable and available than in operando synchrotron-based techniques. 

## 2. Materials and Methods

For the investigation of different structures and their properties, DFT calculations used in this work utilized the Vienna Ab Initio Simulation Package (VASP) [27,28,29]. As part of this package, the Perdew-Burke-Ernzerhof form of the generalized gradient approximation function (GGA-PBE) was combined with the projector augmented wave (PAW) method, including spin polarization [30]. Taking into account dispersion interactions, DFT-D3 corrections were also included in the calculations [31]. The cut-off kinetic energy was set at 600 eV and the width of the Gaussian smearing for all occupied electronic levels was set at 0.2 eV. The first irreducible Brillouin zone was obtained by forming a *Γ*-centered 6×6×1 *k*-points mesh using the general Monkhorst-Pack scheme [32]. Relaxation of the atoms in the simulation cell was enabled until the value of the Hellmann-Feynman forces that acted on them did not drop below 0.01 eV Å^−1^. All of the shown models of the structures in this work were created using the Visualisation for Electronic Structural Analysis program (VESTA) [33].

The starting structure of pyridine-N_4_ graphene was modelled by first forming a di-vacancy in the 4×4 supercell of pristine graphene with 32 C atoms—a defect that was created by removing two neighboring carbon atoms. Then, the four closest carbon atoms were replaced with nitrogen atoms. The model created in this manner had the formula N_4_C_26_. In the next step, one, two, or three of these nitrogen atoms were replaced with either oxygen or sulfur atoms. Finally, systems with metals were obtained by embedding transition metal atoms that showed good results in previous work investigating structures, namely manganese, iron, and cobalt [23].

The embedding energy of the metals, *E*_emb_(M), was calculated as:

*E*_emb_(M) = *E*(M@X_x_N_y_G) − *E*(X_x_N_y_G) − *E*(M). (1)

where *E*(M@X_x_N_y_G) represents the energy of the system that contains metal, *E*(X_x_N_y_G) represents the energy of the di-vacant pyridine-like graphene, and *E*(M) represents the energy of the isolated metal atom, while x and y are natural numbers, such that x + y = 4. It should be noted that X indicates either O or S atoms, depending on the corresponding system. 

Depending on the experimental conditions, different surface functionalization can occur during synthesis, including hydrogen, oxygen, or hydroxyl group adsorption. These adsorption reactions can be represented electrochemically (all written as half-reactions in the direction of reduction):

M*^z^*^+^ + *z*e^−^ + X_x_N_y_G → M@X_x_N_y_G, (2)

M@X_x_N_y_G + H^+^ + e^−^ → H–M@X_x_N_y_G (3)

OH–M@X_x_N_y_G + H^+^ + e^−^ → M@X_x_N_y_G + H_2_O (4)

O–M@X_x_N_y_G + 2H^+^ + 2e^−^ → M@X_x_N_y_G + H_2_O. (5)

The adsorption energy, *E*_ads_(A), which describes bond formation between adsorbates and the investigated systems, was calculated as:

*E*_ads_(A) = *E*_subs+A_ − (*E*_subs_ + *E*_A_) (6)

where the energy of the substrate with the adsorbed atom or group is *E*_subs+A_, the energy of the substrate is *E*_subs_, and the energy of the isolated adsorbate A (A = H, O or OH) is *E*_A_.

In order to examine an additional thermodynamic aspect of the obtained systems, vibrational analysis was also performed. The approach of interrogation of second-order finite differences was used, and the displacements in all three directions were set to 0.015 Å. However, to save some computational time, constrained dynamics was utilized where the displacements were limited to metal atoms, nitrogen/oxygen/sulfur atoms, and carbon atoms that formed a bond with them, i.e., only the first and second coordination spheres of the metal center were considered for vibrational analysis.

The obtained values of the vibrational frequencies were used to calculate thermodynamic functions, such as zero-point energy (ZPE) and vibrational contribution to entropy, TS_vib_. Then, the chemical potential, $\mu_{i}$, was obtained for each phase in electrochemical Equations (2)–(5):

(7) $$\mu_{i}=E_{\mathrm{tot}}+\mathrm{ZPE}-{\mathrm{TS}}_{\mathrm{vib}}$$

where *E*_tot_ represents the energy of the given phase obtained using DFT.

It should be noted that the chemical potential of pure water was calculated in the gas phase at a temperature of 298 K and pressure of 1 atm, and the obtained value was additionally corrected using the Gibbs free energy change corresponding to evaporation under the same conditions as listed.

Based on these results, the Gibbs free energy change for reactions (2)–(5), ∆*G*, was calculated:

(8) $$\Delta G=\overset{}{\underset{i,\;\mathrm{products}}{\sum }}\mu_{i}-\overset{}{\underset{i,\mathrm{reactants}}{\sum }}\mu_{i}$$

The downside of using constrained dynamics can be seen when calculating ΔG—the obtained value was smaller by 0.03–0.05 eV than the value obtained without constraints [23].

In this paper, a hypothetical galvanic cell was used, where the reactions of interest took place on the cathode, while the hydrogen electrode represented the anode. Keeping this in mind and assuming the equilibrium of the hydrogen electrode (the reference potential of the hydrogen electrode was set so that the electrochemical potential of the (H^+^ + e^−^) system was the same as the potential of ½ H_2_ under the following conditions: T = 298 K, p = 1 atm, and pH = 0),

H^+^ + e^−^ → ½ H_2 (g),_ (9)

the standard electrode potentials were calculated (vs. standard hydrogen electrode, SHE) for electrochemical reactions (2)–(5). This was performed by utilizing the computational hydrogen electrode approach [34].

Based on the value of the Gibbs free energy change, the electromotive force for each reaction (2)–(5) was also calculated by simply dividing ΔG by the number of electrons exchanged in the system during the corresponding reaction. Since, as mentioned, the hydrogen electrode represented the anode, the electromotive force was equal to the standard electrode potential of the given reaction. A somewhat different system was considered for the metal embedding reactions—a hypothetical galvanic cell where one electrode was constructed using the transition metal in question and the other was the given M@X_x_N_y_G electrode [23].

Finally, the optical spectra were calculated from the frequency-dependent microscopic polarizability matrix using projector-augmented wave (PAW) methodology [35].

## 3. Results and Discussion

### 3.1. Embedding Co, Fe, and Mn into X_x_N_y_-Graphene

**Figure 1:**
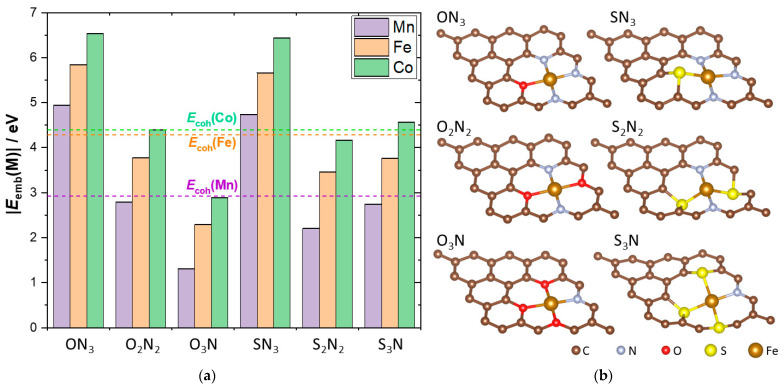
(**a**) Embedding energies of Mn, Fe, and Co into X_x_N_y_-graphene. The composition of the X_x_N_y_-center is given on the *x*-axis. Cohesive energies of the three metals (taken from Ref. [36]) are shown as dashed horizontal lines for comparison; (**b**) optimized structures of Fe@X_x_N_y_G. The composition of the X_x_N_y_ moiety is given above each inset.

Embedding the chosen metal atoms into mixed X_x_N_y_-centers resulted in the formation of M@X_x_N_y_G surfaces. The optimized structures of M@X_x_N_y_G showed similar general characteristics for the same X_x_N_y_-centers for all three metals. As an example, the structures of Fe@X_x_N_y_G are shown in Figure 1b. While the O-containing M@X_x_N_y_G systems remained planar, the S-containing models showed a significant protrusion of S and its first neighbors from the graphene plane. Of course, the M−X bond lengths changed depending on the metal. The average bond lengths between M and its first neighbors are given in Table 1. The corresponding bond lengths changed in the order Mn > Fe > Co, which was attributed to differences in the radii of the metals.

The embedding energies of M@X_x_N_y_G are shown graphically in Figure 1a. In order to evaluate the stability of the M single atoms in X_x_N_y_G, we compared these energies with the cohesive energies of the corresponding metals, E_coh_(M). Co was found to be stable in all of the investigated X_x_N_y_-centers. For ON_3_ and SN_3_ mixed centers, *E*_emb_(M) surpassed E_coh_(M) for all three metals, i.e., the metals were more stable in X_x_N_y_G than in the bulk metal lattice. However, that was not the case for Mn and Fe in other investigated mixed centers, where some metal aggregation was possible. We noticed that for one X_x_N_y_G center, the embedding energies followed the general trend |*E*_emb_(Co)| > |*E*_emb_(Fe)| > |*E*_emb_(Mn)|. The calculated |*E*_emb_(M)| values were generally lower than those of N_4_-graphene [23]. The higher O content in O_x_N_y_ was found to have a weakening effect on *E*_emb_(M@O_x_N_y_G). Finally, we noted that for the X_2_N_2_ coordination environments, there were two symmetrically different possibilities. The possibility considered here (Figure 1b) was formally represented as ONON (SNSN), going clockwise. In the cases of both O and S, this configuration was energetically more stable than ONNO (SNNS, going clockwise again), by 0.461 eV in the case of the O_2_N_2_ environment and 1.835 eV in the case of the S_2_N_2_ environment. Thus, only the X_2_N_2_ arrangement presented in Figure 1b was studied here. 

### 3.2. H, O, and OH Adsorption on M@X_x_N_y_G

**Figure 2:**
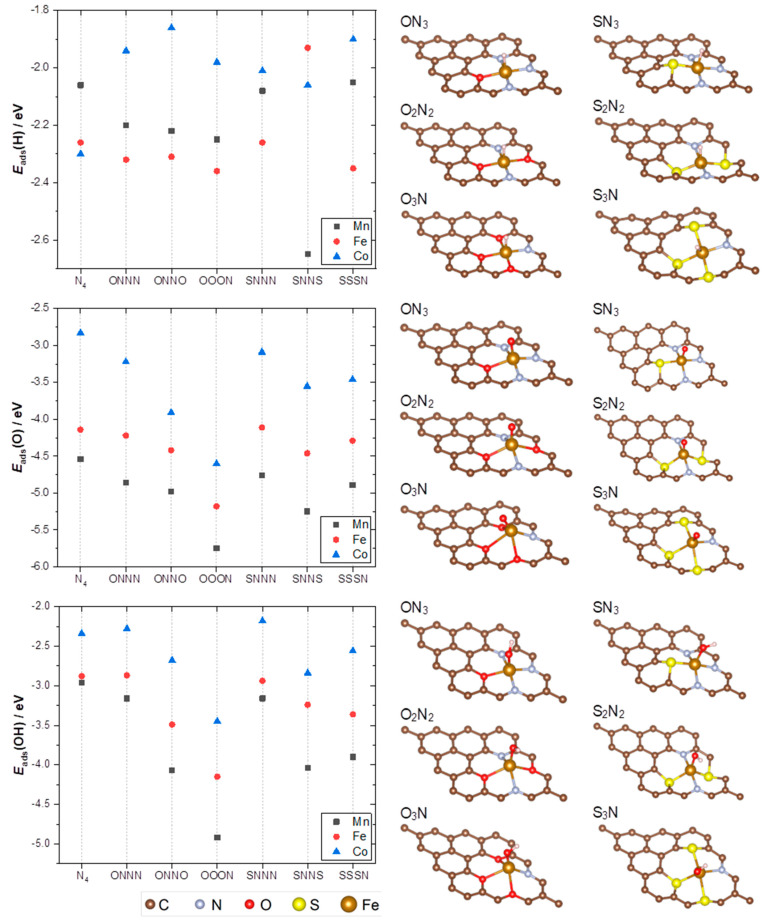
Left: adsorption energies of H (**top**), O (**middle**), and OH (**bottom**) on M@X_x_N_y_-graphene models (M = Mn, Fe, or Co, as indicated in the legend). The composition of the mixed X_x_N_y_-center is given on the *x*-axis. The corresponding adsorption energies on M@N_4_G are given for comparison (values from Ref. [23]). Right: optimized structures of A@Fe@X_x_N_y_-graphene (A = H, O or OH).

Next, we investigated the adsorption of H, O, and OH onto the M@X_x_N_y_G systems shown above at the M-top adsorption site (directly above the metal atom). First, let us focus on the adsorption of atomic hydrogen. The strongest H binding was found in the case of H@Mn@S_2_N_2_G, with *E*_ads_(H) amounting to −2.65 eV. For nearly all of the investigated systems, we found that for the same X_x_N_y_ center, *E*_ads_(H@M@X_x_N_y_G) followed the trend |*E*_ads_(H@Fe@X_x_N_y_G)| > |*E*_ads_(H@Mn@X_x_N_y_G)| > |*E*_ads_(H@Co@X_x_N_y_G)|. Thus, H adsorption was strongest when Fe was embedded in the given center and weakest when Co was embedded (Figure 2). The only exception from this trend was the S_2_N_2_ center, for which H binding was strongest when M was Mn and weakest when M was Fe. Comparing the studied systems with their N_4_-analogues, we found that Co embedded in the mixed center bound hydrogen more weakly than the N_4_-case, while for Mn and Fe, H binding could be stronger or weaker, depending on the nature of the chosen center.

Regarding O adsorption, all of the investigated systems showed stronger O binding than their N_4_ analogs, except for O@Fe@SN_3_G, for which O binding was weaker by approximately 0.03 eV. The strongest O binding was found for O@Mn@O_3_NG, with *E*_ads_(O) amounting to −5.75 eV. As a general trend for O adsorption on O-containing mixed centers in graphene, we found that higher O content in the mixed center yielded stronger O binding onto M for all three investigated metals. On the other hand, for S-containing mixed centers, we found that the strongest O binding corresponded to the M@S_2_N_2_G systems.

Finally, for the case of OH adsorption, the strongest binding was found for OH@Mn@O_3_NG (same system as for O_ads_), with *E*_ads_(OH) equal to −4.92 eV. OH adsorption was found to follow nearly the same trend as O adsorption, with the only difference being that for Fe embedded into S-containing mixed centers, OH binding grew stronger with increasing S content. For all Mn@X_x_N_y_G, OH binding was stronger than on Mn@N_4_G. This was also true for most Fe- and Co-containing centers compared to Fe@N_4_G and Co@N_4_G. While H adsorption induced minimal deformation of M@X_x_N_y_G, adsorption of O and OH caused larger protrusions of M and its surrounding atoms (Figure 2, right).

### 3.3. Thermodynamics & Electrochemistry

We investigated redox pairs M^z+^/M@X_x_N_y_G, M@X_x_N_y_G/H–M@X_x_N_y_G, OH–M@X_x_N_y_G/M@X_x_N_y_G, and O–M@X_x_N_y_G/M@X_x_N_y_G and calculated their standard potentials (E^0^(O/R)) and equilibrium potentials (E(O/R)) at standard conditions and at 298 K. This was performed using ground state energies, ZPE, and entropic contributions for the M@X_x_N_y_G, H−M@X_x_N_y_G, HO−M@X_x_N_y_G, and O−M@X_x_N_y_G systems. The calculated standard potentials (E^0^(O/R)) are summarized in Table 2.

Metal dissolution, described by Equation (2), is not pH-dependent but the adsorption of H, O, and OH are pH-dependent, with the slope of E vs. pH being 59 mV per pH unit:

*E*(M*^z^*^+^/M@X_x_N_y_G) = *E*^0^(M*^z^*^+^/M@X_x_N_y_G) + (0.059 V/*z*) ∙ log *a*(M*^z^*^+^), (10)

*E*(M@X_x_N_y_G/H–M@X_x_N_y_G) = *E*^0^(M@X_x_N_y_G/H–M@X_x_N_y_G) − 0.059 V × pH (11)

*E*(OH–M@X_x_N_y_G/M@X_x_N_y_G) = *E*^0^(OH–M@X_x_N_y_G/M@X_x_N_y_G) − 0.059 V × pH (12)

*E*(O–M@X_x_N_y_G/M@X_x_N_y_G) = *E*^0^(O–M@X_x_N_y_G/M@X_x_N_y_G) − 0.059 V × pH. (13)

We used a(M^z+^) = 10^−8^ mol dm^−3^ and calculated E(O/R) for pH values from 0 to 14, which allowed us to identify the stable phase for each E/pH combination, based on the rule that the most stable oxidized phase is the one with the lowest equilibrium potential, while the most stable reduced phase is the one with the highest equilibrium potential. When we applied this rule at pH = 0, the standard electrode potentials (Table 2) indicated that ON_3_ and SN_3_ were the only X_x_N_y_-centers in which Fe and Co were stable and would not dissolve (*E*^0^(M^z+^/MX_x_N_y_G) > *E*^0^(H^+^/H_2_)). In addition, we noticed a trend that a larger atomic number for M (Z(Mn) < Z(Fe) < Z(Co)) yielded a more stable system (M embedded instead of dissolved). A similar result was previously reported for N_4_-graphene [23].

Since *E*(M^z+^/M@X_x_N_y_G) does not depend on pH, while other equilibrium potentials are pH-dependent, an increase in pH will enable *E*(M^z+^/M@X_x_N_y_G) to be greater than E(M@X_x_N_y_G/H–M@X_x_N_y_G), even though this was not the case at pH = 0. When this is true, systems with adsorbed H are stable. A similar result could occur with adsorbed O or OH. The lowest pH values for which H, O, or OH could cover M are given in Table A1. However, it should be noted that the nature of the most stable phase depends not only on pH but also on the electrode potential. Therefore, this table only provides the pH range for which it is possible to obtain the adsorbed species at any E. In the case of Fe, hydrogen did not cover Fe@O_3_NG, and OH only adsorbed on Fe@ON_3_G and Fe@SN_3_G systems at pH values of 7.29 and 7.81, respectively. Oxygen adsorption was only achieved on one system containing Fe: Fe@ON_3_G at a very high pH (13.61). In the case of manganese, unlike cobalt and iron, hydrogen adsorption only occurred on two systems—Mn@ON_3_G and Mn@SN_3_G. However, the hydroxyl group was adsorbed on four out of six systems and adsorption did not occur on Mn@S_2_N_2_G and Mn@S_3_NG. Oxygen could not be adsorbed on any system.

**Figure 3:**
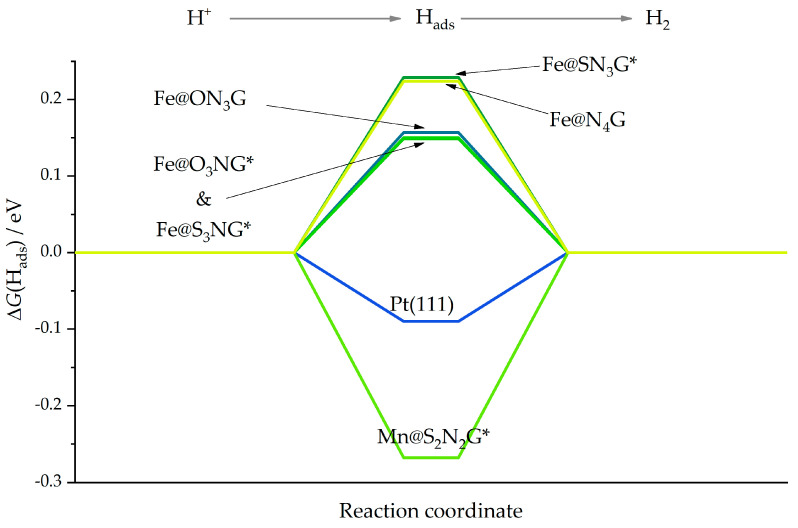
HER reaction profiles for selected systems. Those marked with (*) are not stable at 0 V vs. Reversible Hydrogen Electrode.

The data acquired in previous work [23] and presented here can be used to screen the catalytic performance of studied SACs toward HER. To do this, we followed the work of Nørskov et al. [37] and used calculated ΔG values for H_ads_ formation to construct HER reaction profiles. The data for Pt(111), serving as a reference, were obtained from Ref. [37]. Figure 3 assembles several SACs where low HER overvoltages were observed based on the calculated ΔG(H_ads_) values.

The presented systems with Fe centers showed that the formation of H_ads_ required energy input. Thus, H_ads_ will be formed at potentials below 0 vs. RHE. However, compared to the well-known Fe@N_4_G system, activity improvements were expected for Fe@ON_3_G, Fe@O_3_NG, and Fe@S_3_NG, while Fe@SN_3_G should display very similar HER activity as Fe@N_4_G. Nevertheless, based on the potentials presented in Table 2, the Fe@O_3_NG and Fe@S_3_NG systems should not be stable at 0 V vs. RHE. Thus, the metal center would dissolve in highly acidic media, which corresponded to the considered HER conditions. Moreover, going back to Figure 1, one can see that for the Fe@O_3_NG and Fe@S_3_NG systems, *E*_emb_(Fe) was also below the Fe cohesive energy. This not only means that the metal centers could dissolve under HER conditions, but also that some Fe aggregation could be expected during the synthesis of such SACs. This observation reinforced the conclusion that active center stability must be considered before assessing activity.

**Figure 4:**
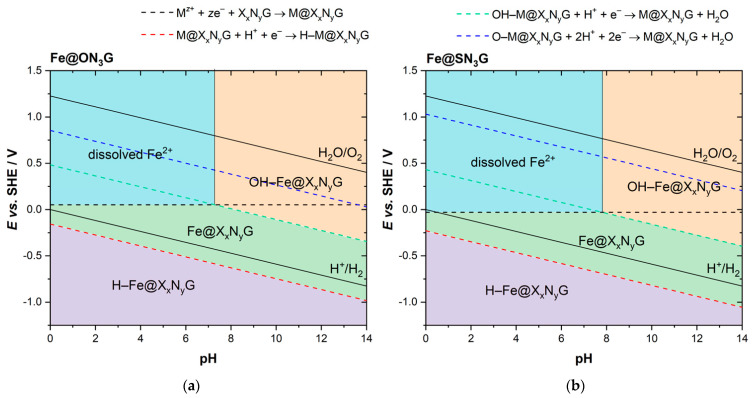
Surface Pourbaix plots for (**a**) Fe@ON_3_-graphene and (**b**) Fe@SN_3_-graphene model systems. Solid black lines (H^+^/H_2_ and H_2_O/O_2_) indicate the theoretical water stability region.

In addition to stability, one should also consider the speciation of the SAC active center under different reaction conditions. Thus, we finally constructed surface Pourbaix diagrams, allowing us to easily determine which phase was the most stable for a given *E* and pH. We chose two systems, Fe@ON_3_G and Fe@SN_3_G (Figure 4), which should have been relatively stable in a wide range of pH values and electrode potentials, based on the data presented in Table 2. In fact, Co and Fe at the ON_3_ and SN_3_ centers were found to be the only stable metal atoms at pH = 0 among all the studied cases. The Fe center was previously found to be stable at the N_4_-center in graphene for all pH values [23]. We found that in the case of the mixed X_x_N_y_ centers, dissolution was possible, but we also found regions where Fe@X_x_N_y_G was stable and where adsorbed H or OH were stable.

**Figure 5:**
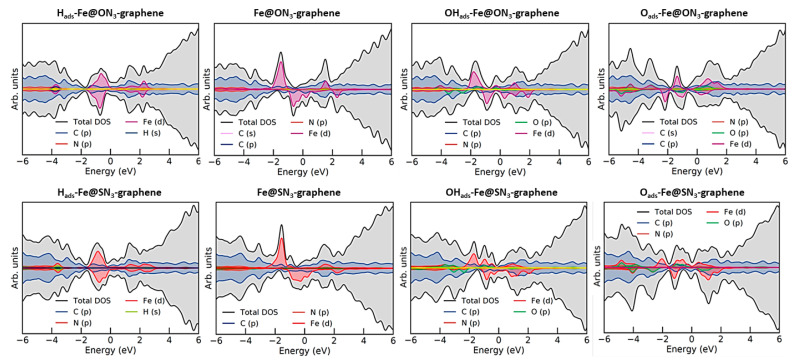
Density of state (DOS) plots for the Fe@ON_3_ system (**top row**) with H_ads_, bare metal center, OH_ads_, and O_ads_, in that order from left to right, and the analogous DOS plots for the Fe@SN_3_ system (**bottom row**). The DOS plots were generated using the Sumo tool for VASP [38].

Considering the results presented in Figure 4, one can see that both systems could easily dissolve at potentials close to 0 V vs. RHE (please note that the Nernst potential for dissolution was calculated from the standard potentials and assuming an Fe concentration (activity) of 10^−8^ mol dm^−3^, which shifts the equilibrium potential to lower values). At pH values above 8, both systems tended to adsorb OH rather than getting dissolved. However, this also meant that under oxygen reduction reaction conditions, both centers should bind OH_ads_ and the reaction was likely to proceed at the active site, which was not bare as in a vacuum. Therefore, natural questions arise regarding the stability and activity of such modified active centers. Such questions were recently tackled by Nematollahi et al. [12], showing that in the case of Fe-N_4_ centers, a fifth ligand such as −NH_2_, −OH, and −SO_4_ can improve stability and direct the oxygen reduction reaction towards the selective 4e reduction pathway. This means that it is necessary to consider other possible adsorbates that can tune the active center’s electronic structure, stability, and reactivity. However, it is more than obvious that the electronic structure of the active center is particularly affected by the presence of different adsorbates (Figure 5). At first glance, for the Fe@ON_3_G and Fe@SN_3_G systems, it seemed that the electronic structures of the bare metal centers were mutually similar and mainly determined by nitrogen ligands. The same held true for centers with H_ads_. However, the situation differed for Fe centers with OH_ads_ and O_ads_, which was reflected in somewhat different oxidation potentials, particularly for cases of O_ads_-Fe. 

### 3.4. Observing Active Center Change

**Figure 6:**
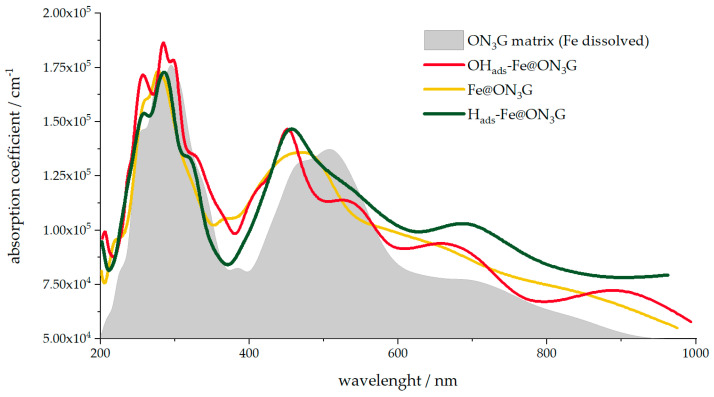
Optical spectra of Fe@ON_3_G SACs (bare, H_ads_-, and OH_ads_-covered centers) and the spectrum of the corresponding ON_3_G matrix, analogous to the case when the metal center is dissolved.

As mentioned, there is direct experimental evidence that SAC active centers might differ from their “pristine” forms under operating conditions. For example, using *in operando* XANES and FT-EXAFS [11], it was shown that under oxygen reduction reaction conditions, OH_ads_ formed at the Fe-N_4_ center. As we previously discussed [23], synchrotron-based techniques are not widely available, particularly in situ and in operando. However, less sophisticated techniques may be sufficient to show that the active center has changed under potential cycling or due to the pH of the environment, or at least demonstrate qualitative changes. We previously suggested in situ FTIR as a possible option, where the characteristic bands could be used to confirm the formation of some adsorbate species at the metal center or carbon matrix [23]. On the other hand, Figure 5 clearly demonstrates that the electronic structure of the SAC near the Fermi level changed upon adsorption of different species. Thus, one might expect that the optical properties of SACs would also change in the case of potential modulation or pH change, leading to the dissolution of metals or adsorption at metallic sites. Figure 6 shows the calculated UVC-Vis-NIR spectra (from 200 to 1000 nm) of bare Fe@ON_3_G systems with H_ads_, O_ads_, and OH_ads_, but also of an empty ON_3_G moiety, corresponding to the situation when the Fe center is expected to dissolve (pH < 7 and potentials above 0 V vs. RHE, Figure 4). We noticed that the use of UV-Vis spectroscopy for the study of porphyrins is rather widespread and the technique is sensitive to the coordination of metal centers [39,40,41].

While the spectra presented in Figure 6 were obtained theoretically, two important points should be emphasized. First, changes in the presented optical spectra (Figure 6) were prominent enough to at least confirm changes in the active site structure. Second, the overall shape of the calculated UV-Vis-NIR spectra was in very good agreement with that of previously reported metalloporphyrins [39,40,41], suggesting that the theory has properly captured the description of the systems. Although optical spectroscopy is not as informative as FTIR and more advanced techniques and thus cannot easily identify the actual structure of the active site, we believe it has certain advantages. Namely, the technique is inexpensive, rather fast, can be performed in situ, and does not have problems associated with the adsorption of incident light by water. Thus, with many existing spectroelectrochemical systems commercially available, using UV-Vis spectroscopy in situ and in operando would be relatively easy, at least to indicate that changes are taking place at the SAC active center. 

## 4. Conclusions

In the present work, we addressed the role of metal center coordination in SACs comprised of Mn, Fe, and Co, which were embedded in O_x_N_y_G or S_x_N_y_G moieties in the graphene basal plane (x + y = 4). Compared to well-known M@N_4_ SACs studied before, the SACs with mixed coordination of metal centers showed altered embedding energies, reactivity, and stability. First of all, for systems with a low number of N atom-coordinating metal centers, the embedding energies were higher than the cohesive energies, suggesting that metal precipitation might occur. However, even if metal precipitation could be avoided, the metal centers were prone to dissolution and showed altered reactivity compared to their M@N_4_ counterparts. We showed that replacing a certain number of N atoms in the Fe coordination shell with O or S can tune the hydrogen binding energy and improve HER activity compared to the original Fe@N_4_ SAC. However, it is important to emphasize that only a few of the systems expected to improve HER activity (relying on descriptor-based estimations) were actually stable at low pH (~0). Other systems are expected to dissolve under HER operating conditions. Thus, one of the main messages is that estimations of catalyst activity should be considered only if the stability is confirmed. Finally, we discussed the possible use of UV-Vis spectroscopy to (at least) confirm changes in the structure of the active site. Namely, UV-Vis spectroscopy has a long history of use in studying porphyrins, whose metallic centers have a similar coordination as the SACs studied here. Moreover, UV-Vis spectroscopy is fast, affordable, and can be used *in operando* to monitor spectral changes associated with altered coordination of the metal center and its electronic structure. Thus, we hope the present theoretical work will motivate further experimental research to look carefully into changes in the SAC active site structure under operating conditions, using characterization techniques with different levels of complexity. 

## Figures and Tables

**Table 1:** Average bond lengths (*d*) between metal M and its first neighbors in M@X_x_N_y_G systems.

M	Systems Containing O		Systems Containing S	
	*d*(M−N)/Å	*d*(M−O)/Å	*d*(M−N)/Å	*d*(M−S)/Å
Co	1.85	1.92	1.91	2.08
Fe	1.87	1.92	1.92	2.12
Mn	1.89	1.94	1.94	2.20

**Table 2:** Calculated standard electrode potentials for reactions in Equations (2)–(5) at *T* = 298 K, pH = 0.

System	*E*^0^ (M*^z^*^+^/MX_x_N_y_G)/V	*E*^0^ (MX_x_N_y_G/H−MX_x_N_y_G)/V	*E*^0^ (OH−MX_x_N_y_G/MX_x_N_y_G)/V	*E*^0^ (O−MX_x_N_y_G/MX_x_N_y_G)/V
Co@ON_3_G	0.356	−0.548	1.133	1.166
Co@O_2_N_2_G	−0.716	−0.596	0.718	0.913
Co@O_3_NG	−1.455	−0.452	−0.137	1.070
Co@SN_3_G	0.304	−0.450	1.204	1.211
Co@S_2_N_2_G	−0.845	−0.409	0.604	1.380
Co@S_3_NG	−0.617	−0.579	0.802	1.332
Fe@ON_3_G	0.288	−0.157	0.482	0.855
Fe@O_2_N_2_G	−0.735	−0.174	−0.118	1.266
Fe@O_3_NG	−1.487	−0.149	−0.859	1.165
Fe@S_3_G	0.206	−0.229	0.431	1.031
Fe@S_2_N_2_G	−0.893	−0.564	0.200	0.916
Fe@S_3_NG	−0.731	−0.150	−0.030	1.286
Mn@ON_3_G	−0.228	−0.301	0.161	0.533
Mn@O_2_N_2_G	−1.296	−0.256	−1.077	1.668
Mn@O_3_NG	−2.037	−0.215	−1.635	1.369
Mn@SN_3_G	−0.314	−0.415	0.191	0.641
Mn@S_2_N_2_G	−1.602	0.268	−0.700	0.991
Mn@S_3_NG	−1.298	−0.482	−0.562	1.217

## Data Availability

Data are available upon reasonable request.

## Appendix A

**Table A1:** The lowest pH at which H, O, and OH can be found adsorbed on M@X_x_N_y_G. Dash marks indicate that the given species was not found on M (as the most stable phase) for any pH value.

System	pH_min_ (H−M@X_x_N_y_G)	pH_min_ (OH−M@X_x_N_y_G)	pH_min_ (O−M@X_x_N_y_G)
Co@ON_3_G	0.00	-	-
Co@O_2_N_2_G	6.03	-	-
Co@O_3_NG	-	-	-
Co@SN_3_G	0.00	-	-
Co@S_2_N_2_G	11.39	-	-
Co@S_3_NG	4.64	-	-
Fe@ON_3_G	0.00	7.29	13.61
Fe@O_2_N_2_G	13.51	-	-
Fe@O_3_NG	-	-	-
Fe@SN_3_G	0.00	7.81	-
Fe@S_2_N_2_G	9.58	-	-
Fe@S_3_NG	13.85	-	-
Mn@ON_3_G	2.76	10.59	-
Mn@O_2_N_2_G	-	7.71	-
Mn@O_3_NG	-	10.81	-
Mn@SN_3_G	2.23	12.56	-
Mn@S_2_N_2_G	-	-	-
Mn@S_3_NG	-	-	-
